# Increased risk of internal tumors in DNA repair-deficient xeroderma pigmentosum patients: analysis of four international cohorts

**DOI:** 10.1186/s13023-022-02203-1

**Published:** 2022-03-04

**Authors:** Sergey Nikolaev, Andrey A. Yurchenko, Alain Sarasin

**Affiliations:** 1 INSERM U981, Gustave Roussy Institute, Université Paris-Saclay, Villejuif, France; 2 Laboratory of Genome Integrity and Cancers, CNRS UMR9019, Gustave Roussy Institute, Université Paris-Saclay, 114 rue Edouard Vaillant, 94805 Villejuif, France

**Keywords:** Nucleotide excision repair, Transcription-coupled repair, Leukemia, UV-light, Internal cancers

## Abstract

**Background:**

Xeroderma pigmentosum (XP) is a rare, autosomal, recessive DNA repair-deficiency disorder with a frequency of 1–3 per million livebirths in Europe and USA but with higher frequencies in isolated islands or in countries with a high level of consanguinity. XP is characterized by high incidence of skin cancers on sun-exposed sites. Recent improvement in life expectancy of XP patients suggests an increased risk of frequently aggressive and lethal internal tumors. Our purpose was to quantify relative risks of internal tumor development for XP patients by tumor type, XP-subtype, patients’ ages and ethnicity through comparison with the US general population.

**Methods:**

We analyzed four independent international well-characterized XP cohorts (from USA, UK, France and Brazil) with a total of 434 patients, where 11.3% developed internal tumors and compared them to the American general population. We also compiled, through PubMed/Medline, a dataset of 89 internal tumors in XP patients published between 1958 and 2020.

**Results:**

In the combined 4-XP cohort, relative risk of internal tumors was 34 (95% confidence interval (CI) 25–47) times higher than in the general population (*p*-value = 1.0E−47) and tumor arose 50 years earlier. The XP-C group was at the highest risk for the 0–20 years old-patients (OR = 665; 95% CI 368–1200; *p*-value = 4.3E−30). The highest risks were observed for tumors of central nervous system (OR = 331; 95% CI 171–641; *p*-value = 2.4E−20), hematological malignancies (OR = 120; 95% CI 77–186; *p*-value = 3.7E−36), thyroid (OR = 74; 95% CI 31–179; *p*-value = 1.2E−8) and gynecological tumors (OR = 91; 95% CI 42–193; *p*-value = 3.5E−12). The type of mutation on the *XPC* gene is associated with different classes of internal tumors. The majority of French XP-C patients (80%) are originated from North Africa and carried the *XPC* delTG founder mutation specific from the South Mediterranean area. The OR is extremely high for young (0–20 years) patients with more than 1300-fold increase for the French XPs carrying the founder mutation.

**Conclusion:**

Because the age of XP population is increasing due to better sun-protection and knowledge of the disease, these results are of particular importance for the physicians to help in early prevention and detection of internal tumors in their XP patients. Few preventive blood analyses or simple medical imaging may help to better detect early cancer appearance in this population.

**Supplementary Information:**

The online version contains supplementary material available at 10.1186/s13023-022-02203-1.

## Introduction

Xeroderma pigmentosum (XP) is an autosomal recessive disease, caused by deficient nucleotide excision repair (NER) [1–3]. XP is rare with an incidence of 1–3/1,000,000 in Europe [4] and USA [5], while it is more common in some countries such as Japan [6], Pakistan [7] and Comoros [8]. One of the highest frequencies of XP patients (> 100/1,000,000) is reported in North Africa and associated with high allelic frequency of a causative mutation in the population and with traditions of consanguinity [9]. Characteristic findings of XP include photosensitivity, actinic keratosis, cutaneous atrophy and early onset of cutaneous tumors [8, 10–13]. There are seven complementation groups that are involved in the classical XP disease, caused by bi-allelic mutations in one of *XPA, B, C, D, E, F, G* genes [12, 14]. The XP Variant (XP-V) is NER-proficient but is caused by mutations in the *POLH* gene coding the translesion DNA polymerase-η [15, 16].

NER is involved in repair of various bulky adducts besides UV-induced-photoproducts, such as those induced by genotoxins in cigarette smoke, genotoxic food contaminants or ROS-induced DNA damage [12]. We hypothesize that NER deficiency might also be a cause of increased risk of non-skin cancers. Indeed, a 12-fold increased frequency of developing internal neoplasms in XP was reported in the past [17]. In line with that, we described 25-fold increased mutation rates in leukemia developed by XP patients with a characteristic mutational profile [18].

In order to quantify the risk of internal tumors in the XP population, we analyzed data from 4 international clinically well-characterized XP cohorts. In parallel, we conducted a systematic research investigating all published reports on internal tumors (non-skin cancers) in XP patients since 1958.

## Method

### Data sources

We searched PubMed for “xeroderma pigmentosum” and analyzed papers describing non-redundant internal tumors. The first paper appeared in 1958 [19] and the latest is this one. We defined as internal tumors all described malignant tumors in XP patients except those due to sun exposure such as skin cancers. Lip and tongue tumors and cutaneous angiosarcoma have also been removed because they are partially linked to sun exposure [8]. We retrieved clinical descriptions, complementation groups and tumor characteristics in 88 XP patients (89 tumors) [20–45].

### French cohort

Among 176 XP patients diagnosed in our laboratory, we already described 23 internal tumors [18, 28, 29, 35, 42] and 9 new patients are reported here. All patients were followed in University hospitals in France and sometimes in North Africa. Skin biopsies or blood samples were sent to the Laboratory of DNA repair-deficient diseases at Gustave Roussy (Villejuif, France) or to the Hematology Center at Saint-Louis Hospital (Paris, France) for molecular diagnosis. DNA repair activities, determination of XP complementation groups, Sanger sequencing of XP genes were done as already published [8].

The French cohort is composed of 64% XP-C patients (80% of them are originated from North Africa), 18% are XP-V, 8.5% are XP-D, 7.5% are XP-A, 2 patients are XP-E, one patient is XP-F and one is XP-G.

Written informed consent was provided by patients or their relatives in accordance with the Declaration of Helsinki and French law. This study was approved by the Institutional Review Board of the University Institute of Hematology (IUH; Saint-Louis Hospital, Paris, France), the French Agency of Biomedicine (Paris) (Arrêté n°2001/904 and Ref: AG08-0321 GEN of 27/09/2008; www.agence-biomedecine.fr/Genetique) and the European Commission “Geneskin: Genetics of human genodermatosis” (Brussels, Belgium).

### Brazilian, English and American cohorts

English (89 XPs) and Brazilian (32 XPs) cohorts were published [39, 44]. Up-to-date information concerning the American NIH xeroderma pigmentosum cohort [34, 45] (137 XPs) is a personal communication of Dr. K.H. Kraemer (NIH, Bethesda, USA).

## Results

### Risk of development of internal tumors in XP patients

#### Cohorts of XP patients

We performed a meta-analysis and systematic review of 4 independent XP cohorts: 137 patients in the American NIH cohort (A-XP) with 14 internal tumors (10.2%); 176 patients from France (F-XP) with 32 internal tumors (18.2%); 32 patients from Brazil (B-XP) with 2 internal tumors (6.3%) and 89 patients in the English cohort (UK-XP) with 2 internal tumors (2.2%) (Table 1; Additional file 1: Figure S1A). These cohorts have been independently set up to follow XP patients in terms of clinical, genetic and epidemiological studies without any bias concerning internal tumors.

**Table 1:** Distribution of internal XP tumors and complementation groups* among the four independent XP cohorts

Tumor types^‡^	XP cohorts^†^			
	A-XP (137) (delTG)^$^	F-XP (176) (delTG)^$^	B-XP (32) (delTG)^$^	UK-XP (89) (delTG)^$^
Breast	0	1	0	0
CNS	4	3 (3)	0	2
GI	0	1XP-V	1 XP-V	0
HEM	4 (2)	17 (17)	0	0
KI	0	1 (1)	0	0
LU	3	0	0	0
THY	2 (incl. 1 XP-E)	3 (3)	0	0
Female	1	5 (5)	1 (1)	0
Male	0	1 XP-V	0	0
Total	14 (2)	32 (29)	2 (1)	2 (0)

^*^All these internal tumors occurred in XP-C patients except for 4 patients indicated in the Table

^†^A-XP, F-XP, B-XP and UK-XP refer to the American, French, Brazilian and English XP cohorts (see Methods)

^‡^CNS refers to central nervous system, GI to gastro-intestinal, HEM to hematological malignancies, KI to kidney, LU to lung, THY to thyroid tumors, “Female” means tumor of the woman reproductive system and “Male” tumor of the man reproductive system

^$^Number of XP patients carrying the founder delTG *XPC* mutation from North Africa [13]

#### Risk of internal tumors in the 4-combined XP cohort

**Fig. 1:**
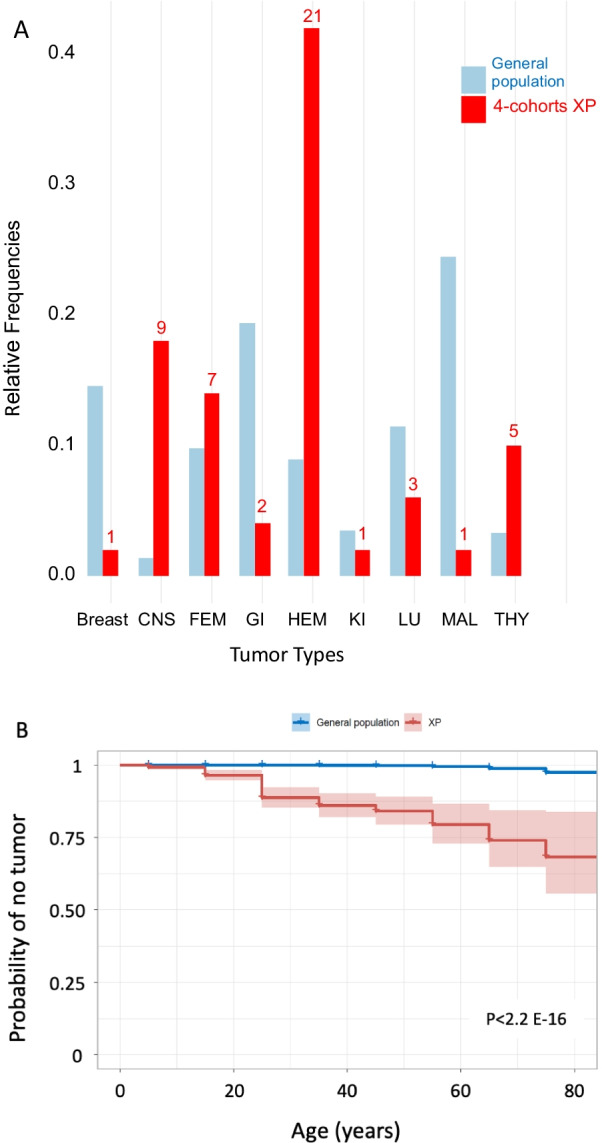
Characteristics of XP patients reported in 4 independent XP cohorts. **A**. Spectrum of tumor types in the 50 tumors versus general population. Relative frequencies of XP internal tumor types (excluding skin cancers) in the grouped 4 XP cohorts (red) as compared to the general American population (blue). The red numbers correspond to the raw counts of XP tumors. One patient had two independent tumors. **B**. Kaplan–Meier plot with 95% confidence intervals of the age of internal tumor onset in XP population versus the general population. The *p*-value is given as compared to the American general population

To estimate frequencies and risks of internal tumors in XP population, we combined all 4-aforementioned cohorts into a unique one consisting of 434 XP patients and including 50 internal tumors (for 49 XP patients). The XP patients develop internal tumors much more frequently than the American general population (11.3% vs 0.47%, *p*-value = 3.4E−58; binomial test, two-sided) (Additional file 1: Figure S1A). The tumor spectrum in XP is characterized by an excess of hematological malignancies (HEM), central nervous system (CNS), thyroid (THY) and gynecological (FEM) tumors as compared to the general population (Fig. 1A). Ages of onset of internal tumors in combined XP cohort is significantly lower than in the general population (Fig. 1B).

The Odds ratio of developing internal tumors (excluding the tumors of reproductive systems that are treated separately by taking into account the gender information) for the 434 XP-cohort is 34 (95% CI = 25–47; *p*-value = 1.0E−47) (Table 2). The particularly strong risks are observed for CNS, HEM, THY and FEM tumors (OR from 74 to 331; Table 2). Age stratification reveals highest risks of tumor onset at young ages of XP patients (0–20 years; OR: 665) (Table 2).

**Table 2:** Risk (OR) of internal tumors according to tumor types, ages and complementation groups following combined analysis of the 4 XP cohorts and of only 3 cohorts excluding the French one (biased for the delTG *XPC* mutation) as well as the French cohort alone

	4 cohorts			3 cohorts (French cohort excluded)			French cohort			French/other 3 cohorts
	OR	95% CI	*p*-value	OR	95% CI	*p*-value	OR	95% CI	*p*-value	OR_F/OR_3cohorts
All internal tumors	34	25–47	1.0E−47	21	13–35	4.9E−16	56	37–84	3.6E−35	2.64
CNS	331	171–641	2.4E−20	371	165–834	2.7E−14	272	87–853	2.3E−07	0.73
HEM	120	77–186	3.7E−36	37	14–99	5.4E−06	253	154–418	7.5E−35	6.84
THY	74	31–179	1.2E−08	49	12–199	8.1E−04	111	35–346	3.4E−06	2.24
FEM*	91	42–193	3.5E−12	50	6–187	8.0E−4	135	53–329	6.9E−10	2.7
MAL*	9	1.3–69	0.10	–	–	–	10	0.24–55	0.1	–
Age 0–20	665	368–1200	4.3E−30	266	84–841	2.5E−07	1330	654–2701	1.9E−25	5.00
Age 21–40	234	153–358	1.5E−47	132	64–271	6.0E−15	381	221–655	2.6E−35	2.89
Age 41–60	22	8–60	4.8E−05	25	8–82	3.0E−04	16	2–116	6.5E−02	0.62
Age 61–80	7	2–30	3.6E−02	10	2–44	1.9E−02	–	–	–	–
XP-C	64	45–90	3.9E−54	42	24–74	3.9E−18	90	58–140	1.6E−38	2.13
XP-E	36	5–281	3.1E−02	46	6–372	2.5E−02	–	–	–	–
XP-V	11	3–47	1.4E−02	12	2–91	8.1E−02	11	1–78	9.2E−02	0.87

^*^We had no access to the gender of XP patients from the UK cohort. Calculations have been made by using the three other XP cohorts

Same legend as in Table 1

XP-C complementation group represents 54% of the 434 XPs-cohort, however 94% of the internal tumors occurred in XP-C (XP-C vs. non-XP-C; OR = 9.8, 95% CI: 3.5−38.1; two-sided Fisher test *p*-value = 3.4E−8) (Table 1).

#### Risk of internal tumors in the French cohort with the delTG XPC mutation (from North African origin) vs the other XP populations

The French XP cohort is composed of 176 patients including 113 XP-C. The vast majority (80%) originated from North Africa, belonged to consanguineous families and shared the same founder mutation: c.1643_1644 delTG; p.Val548AlafsX572 (called here *XPC* “delTG”). We previously estimated that this mutation appeared around 1250 years ago in North Africa indicating that all these patients should have common ancestors [13].

Eighteen % of this French cohort (31 patients for 32 tumors) developed internal tumors (Table 1), demonstrating a 56-fold increased risk as compared to the general population (*p*-value = 3.6E−35) (Table 2). Among these 31 patients, 2 are XP-V (6.5%) and 29 are XP-C (93.5%) among which 28 (90%) originated from North Africa, and carry the *XPC* delTG mutation: 59% are hematological malignancies, 17% gynecological tumors, 10% THY tumors and 10% CNS tumors (Table 1). Genetic homogeneity of the French XP cohort and this high risk of internal tumors might introduce bias in the estimates of risks of internal tumors in the overall XP population. To control for this possibility, we reproduced all the previous analyses on the 3-combined cohorts after excluding the French one: all the results concerning the increased risk of internal tumors in XP are still observed. In the 3-combined cohorts (without the F-XP cohort) the highest cancer risks are again observed for CNS, HEM, THY and FEM tumors (OR: 37–371); the most important risks are again for the young XPs and particularly the XP-C group. At the same time, the French XP-Cs with the delTG mutation exhibit greater risks for all categories of tumors and ages, except for CNS tumors, which show lower Odds ratios than in the pooled 3 cohorts (Table 2).

#### Risk of internal cancer in each independent XP cohort

The Odds ratios of developing internal tumors (excluding the tumors of reproductive systems) in comparison to the general population are 56 (95% CI = 37–84) for F-XP, 33 (95% CI = 19–59) for A-XP, 10 (95% CI = 1.4–73) for B-XP and 7 (95% CI = 1.8–30) for UK-XP (Additional file 1: Figure S2A). This confirms the previous combined data analysis that the French XP-C are at a very high risk of developing internal tumors.

We stratified the XP cases by tumor types, ages and XP complementation groups, and revealed a particularly increased tumor risk in some categories. Odds ratios for CNS tumors are the highest in three cohorts: 469 for A-XP, 361 for UK-XP and 272 for F-XP. The Odds ratios for hematological malignancies are 253 for the F-XP cohort and 71 for the A-XP cohort. The risk for thyroid tumors is also high in F-XP and A-XP (111 and 94, respectively) (Additional file 1: Figure S2B) (95% CI and *p*-values are indicated in the Additional file 1: Figure S2). XP patients aged 0–20 years old exhibit an Odds ratio of 1330 for the F-XP and 594 for the A-XP (Additional file 1: Figure S2C). XP-C patients are at very high risk, particularly in the F-XP, A-XP and the UK-XP cohorts. They demonstrate the highest Odds ratios for developing CNS tumors and hematological malignancies in F-XP and A-XP cohorts (Additional file 1: Figures S3A, B).

The Odds ratios for the tumors of the reproductive systems are also high for the female F-XP (135), A-XP (32) and B-XP (120) corresponding mainly to uterine tumors; while the risk for male-related tumors was not statistically significant compared to the general population (Table 2 and Additional file 1: Figure S3C) (95% CI and *p*-values are indicated in the Additional file 1: Figure S3).

#### Description of all XP patients with internal tumors reported in the literature

Additionally, case reports were published describing XP patients with internal tumors. Following PubMed search, we established an exhaustive list of 89 internal tumors developed by 88 XP patients that corresponds to 79 tumors reported in the literature starting from 1958 up to 2020, one unreported tumor indicated to us by Dr. K.H. Kraemer for the American cohort (NIH, Bethesda, USA) and 9 unpublished tumors from the French XP cohort (reported here in Table 3 and Additional file 1: Table S1). The distributions of these patients according to gender and country of origins are indicated in Additional file 1: Tables S2 and S3. Of course, the 50 internal tumors described in the 4 well-characterized XP cohorts are part of these 89 reported internal tumors.

**Table 3:** All reported xeroderma pigmentosum patients with internal tumor*

Source (years of follow-up, city, country)	No. of XP patients with internal tumor/no. of reported XP patients in the publications	Countries of origin of described XP patients	Genotype of XP patients with internal tumors	Type of internal tumor^†^ (age at diagnosis, sex, country, cell code)	Additional clinical information
Berlin and Tager, (1953–1958, Tel Aviv) and Yosipovitch et al. (1955–1963, Jerusalem, Israel) [19, 20]	1/25	4 countries of Middle East	Med Basin^‡^	Myeloid leukemia (32y, M, Iraq)	Death at 35y
Reed et al.,1969 (Los Angeles, United States) [21]	1/5	United Kingdom	XP-A or XP-D^$^	Acute lymphatic leukemia (3y,M)	Death at 6y
Kraemer et al.,1984 and Kraemer et al.,1987 (1874–1982, Bethesda, United States) [10, 17]	14/830	41 different countries	NR^||^	Astrocytoma (9y, M, Japan)	
			NR	Medulloblastoma (14y, M, NIH^¶^)	
			NR	Brain sarcoma (16y, M, NIH)	
			NR	Brain sarcoma (33y, M, NIH)	Death at 35y
			*XPC*: c.621_622ins83	Bronchogenic carcinoma (34y, M, NIH, XP3BE)	Death at 37y
			NR	Bronchogenic carcinoma (62y, M, France)	
			NR	Pancreatic adenocarcinoma (47y, F, Spain)	
			NR	Breast cancer (38y, F, NIH)	
			NR	Throat cancer (65y, M, NIH)	
			NR	Gastric cancer (67y, M, France)	
			NR	Testicular cancer (12y, M, NIH)	
			NR	Gingival squamous cell (9y, F, NIH)	
			NR	Gingival tumor (17y, F, NIH)	
			NR	Palate squamous cell (18y, M, NIH)	
Puig et al.,1985 (Spain) [22]	1/1	Spain	NR	Gastric adenocarcinoma (30y, F)	Death at 31y
Satoh and Nishigori, 1988 (1975–1987, Kyoto, Japan) [23]	6/272	Japan	XP-A	Glioblastoma (8y, F)	Death at 9y
			XP-F	Bile duct carcinoma (60y, F)	Death at 65y
			XP-V	Transitional cell carcinoma of bladder (68y, M)	Death at 68y
			XP-V	Stomach carcinoma (53y, M)	Death at 53y
			XP-V	SCC of pharynx (51y, M)	Death at 56y
			NR	Uterine carcinoma (49y, F)	Death at 51y
Berbis et al.,1989 (Bordeaux, France) [24]	2/2	Algeria	*XPC*: delTG^#^	RAEB-2 (24y, F, XPGaAiVI)	Death at 25y
			*XPC*: delTG	RAEB-t (27, M, XPGaMVI)	Death at 27y
Tomas et al.,1989 (Spain) [25]	1/1	Spain	NR	Renal leiomyosarcoma (12y, F)	Death at 13y
Salob et al.,1992 (London, UK) [26]	1/1	Pakistan	XP-C	Aplastic anemia as pre-MDS (10y, F)	NR
Visweswara et al.,1997 (Benghazi, Libya) [27]	2/2	Libya	Med Basin	Wilm’s tumor (17y, F)	Death at 18y
			Med Basin	Wilm’s tumor (16y, F)	Death at 17y
Giglia et al.,1998 and Giglia et al.,1999 (Villejuif, France) [28, 29]	3/19	France and North Africa	*XPC*: delTG	Anaplastic astrocytoma (7y, M, Tunisia, XP233VI)	Death at 8y
			*XPC*: delTG	Neuroendocrine thyroid tumor (18y, F, Algeria, XP148VI)	Death at 19y
			XP-V	Gastric adenocarcinoma (48y, F, France, XPGAVI)	Death at 54y
Khatri et al.,1992 and Khatri et al.,1999 (1981–1994, Tripoli, Libya) [30, 31]	2/42	Libya	Med Basin	Follicular carcinoma of thyroid (17y, F)	NR
			Med Basin	Lymphatic leukemia (16y, M)	Death at 18y
Leite et al.,2009 (Sao Paulo, Brazil) [32]	1/3	Brazil	*XPC*: delTG	T-cell lymphoma (3y, M, XP04SP)	Death at 13y
Khan et al.,2006 and Bradford et al.,2011 (1971–2009, Bethesda, United States) [33, 34]	6/106	Different countries	XP-C	Glioblastoma (M, NIH, XP15BE)	Death at 16y
			*XPC*: c.622-2A > C	Spinal cord astrocytoma (22y, M, Native American, XP23BE)	Death at 31y
			*XPC*: c.633-2A > G and Arg155X	Glioblastoma (29y, F, Hungarian, XP24BE)	Death at 35y
			*XPC*: IVS5.1-2A > G	Schwannoma (M, NIH, XP14BE)	Death at 73y
			*XPC:* c.1132_1133delAA	Uterine adenocarcinoma (F, NIH, XP1BE)	Death at 49y
			*XPC*: delTG	Infiltrative pontine astrocytoma (9y, M, Tunisia, XP664VI)	Death at 10y
Hadj-Rabia et al.,2013 (Paris and Villejuif, France) [35]	4/31	North Africa	*XPC*: delTG	T-ALL and AML-6 (12y and 15y, M, Morocco, XP924VI)	Death at 15y
			*XPC*: delTG	Kidney adenocarcinoma (23y, F, Morocco, XP165VI)	Death at 25y
			*XPC*: delTG	Cervical sarcoma (18y, F, Morocco, XP269VI)	Death at 23y
			*XPC*: delTG	Papillary thyroid carcinoma (18y, M, Algeria, XP802VI)	Alive
Janjetovic et al.,2013 (Hamburg, Germany) [36]	1/1	Germany	XP-D	Acute megakaryoblastic leukemia (33y, M)	Death at 34y
Jerbi et al., 2016 (2006–2013, Tunis, Tunisia) [9]	5/64	Tunisia	*XPC*: delTG	Thyroid carcinoma (13y)	Death at 15y
			*XPC*: delTG	Thyroid carcinoma (15y)	Death at 29y
			*XPC*: delTG	Uterine leiomyosarcoma (19y, F)	Alive
			*XPC*: delTG	Uterine leiomyosarcoma (28y, F)	Death at 29y
			*XPC*: delTG	Leukemia (9y, F)	Death at 10y
Pintens et al.,2016 (Brussels, Belgium) [37]	2/2**	Morocco	Med Basin	RAEB and AML (28y, F)	Death at 28y
			Med Basin	ALL (22y, F)	Death at 25y
Lahlimi et al.,2016 (Morocco) [38]	1/1	Morocco	Med Basin	Nephroblastoma (5y, M)	NR
Fassihi et al., 2016 (2010–2016, London, UK) [39]	2/89	Numerous countries	*XPC*: p.Arg220X	Glioblastoma multiforme (38y, Middle East, XP21BR)	Death at 39y
			*XPC:* p.Glu726X	Dysembryonic neuroepithelial tumor (21y, M, Bangladesh, XP28BR)	NR
Coulombe et al.,2016 (Paris, France) [40]	1/1	Zimbabwe	*XPC*: IVS12-1G > C	Gingival squamous cell carcinoma (8y,F)	Death at 10y
Zhang et al.,2018 (Shanghai, China) [41]	1/2^††^	China	*XPC*: p.R718X and p.K431EfsX21	Malignant fibrohistiocytoma (20y, M)	NR
Sarasin et al., 2019 (1983–2015, Villejuif and Paris, France) [42]	10/161	North Africa and Spain	*XPC*: delTG	AML-4 (27y, M, Morocco, XP10VI)	Death at 28y
			*XPC*: delTG	AML-6 (16y, M, Tunisia, XP82VI)	Death at 18y
			*XPC*: delTG	AML-6 (24y, F, Tunisia, XP235VI)	Death at 29y
			*XPC*: delTG	B-ALL and MDS (7y, F, Morocco, XP309VI)	Death at 10y
			*XPC*: delTG	RAEB-2 (24y, F, Spain, XP185VI)	Death at 25y
			*XPC*: delTG	RAEB-t (25y, M, Algeria, XP167VI)	Death at 26y
			*XPC*: delTG	AML (23y, M, Tunisia, XPAHVI)	Death at 25y
			*XPC*: delTG	T-ALL (21y, F, Morocco, XP673VI)	Death at 22y
			*XPC*: delTG	AML (29y, M, Algeria, XP538VI)	Death at 29y
			*XPC*: delTG	AML-6 (29y, F, Morocco, XP2006VI)	Alive after HSCT
Oetjen et al., 2019 (Bethesda, United States) [43]	4/4	North Africa and NIH	*XPC*: delTG	Diffuse large B-cell lymphoma (29y, M, North Africa, XP393BE)	Death at 29y
			*XPC*: delTG	Mixed phenotype acute leukemia (19y, F, Morocco, XP540BE)	Alive at 21y
			*XPC*: delTG and c.1103_1104delAA	MDS and AML (36y, M, NIH, XP30BE)	Death at 38y
			*XPC:* c.622-2A > C	MDS (18y, M, NIH, XP243BE)	Death at 20y
Santiago et al., 2020 (2009–2015, Sao Paulo, Brazil) [44]	2/32	Brazil	*XPC*: delTG	Serous ovary carcinoma (27y, F, 19P0)	Alive
			*XP-V*: c.571 A > C	Gastric adenocarcinoma (50y, M, 2P0)	Death at 54y
Yurchenko et al., 2020 (Villejuif, France) [18]	2/2	Algeria and Comoros	*XPC*: delTG	Uterine rhabdomyosarcoma^$$^,^|| ||^ (16y, F, Algeria, XP2004VI) and AML (22y)	Death at 23y from AML
			*XPC:* IVS12-1G > C	Breast cancer (30y, F, Comoros, XPMYVI)	Death at 30y
Nikolaev and Sarasin, This paper (2015–2020, Villejuif, France)	9/176^¶¶^	North Africa and France	*XPC*: delTG	Uterine rhabdomyosarcoma^|| ||^ (16y, F, Algeria, XP2003VI)	Alive
			*XPC*: delTG	Thyroid carcinoma (17y, F, Algeria, XPAAVI)	Alive
			*XPC*: delTG	Cerebellar astrocytoma (14y, M, Morocco, XPAdSaVI)	Death at 19y
			*XPC*: delTG	Ovarian sarcoma (18y, F, Morocco, XPElHaVI)	Death at 22y
			*XPC*: delTG	Uterine adenomyosarcoma (15y, F, Morocco, XPElKaVI))	Alive
			*XPC*: delTG	Mediastinal T lymphoma (8y, M, Algeria, XP208VI)	Death at 17y
			*XPC*: delTG	AML-3 (14y, M, Morocco, XPMaAbVI)	Alive
			*XPC*: delTG	NK lymphoma (24y, F, Morocco, XP420VI)	Death at 25y
			XP-V: p.Val221ProfsX2	Prostate cancer (60y, M, France, XP819VI)	Alive
Kraemer et al. Personal Communication, 2020 and [45] (Bethesda, United States)	3/137^##^	NIH	*XPC*	Lung cancer (58y, F)	Alive
			*XPC*	Papillary thyroid carcinoma (36y, F, XP570BE))	Alive
			*XPE*	Papillary thyroid carcinoma (57y, F, XP437BE)	Alive
Total of XP patients	88			89 tumors on internal organs	

HSCT, hematopoietic stem cell transplant; RAEB, refractory anemia with excess of blasts; MDS, myelodysplasia (previously called RAEB); AML, acute myeloid leukemia; ALL, acute lymphoblastic leukemia; NR, not reported

*By definition, internal tumors are all tumors except skin tumors (Basal Cell Carcinoma, Squamous Cell Carcinoma, malignant melanoma) and their metastases as well as cutaneous angiosarcoma. We have excluded tongue and lip tumors, which are essentially caused by sun exposure in XP patients

^†^The tumor types are given as reported in the original publications

^‡^We called “Med Basin” XP patients originated from the south part of the Mediterranean see, characterized by early skin cancer development and an absence of neurological deterioration. These patients probably belong to the XP group C and have all chance to exhibit the North African *XPC* founder mutation described as “delTG” (see below and [13])

^$^This patient was described as De Sanctis-Cacchione syndrome, which is mainly associated with *XPA* mutations but can be eventually mistaken with XP-D patient

^||^NR: not reported. At the time of the publications, XP genes were unknown

^¶^These patients were seen at NIH (Bethesda, United States) by the group of K.H. Kraemer. They are probably of American origins but this is not explicitly indicated in the publications

^#^delTG refers to the founder mutation found in the vast majority of XP-C patients from North Africa (Morocco, Algeria, Tunisia, Libya): c.1643_1644delTG; p.Val548AlafsX572 [9, 13]

**Sisters

^††^Monozygotic twins

^$$^This patient developed two different unrelated internal tumors

^|| ||^Monozygotic twins

^¶¶^The total number of patients indicated here (176) corresponds to 161 XP patients described already by us [42] where only hematological malignancies were reported, but where some of these XP patients had also other internal tumors but not reported, plus 15 new XP patients since this last publication

^##^The NIH American cohort of 137 XP patients has 14 individuals with internal tumors. Thirteen were already published and the publications are indicated in the table. One new patient with lung cancer was indicated to us by Dr. K.H. Kraemer and his group as a personal communication

The tumor spectrum in the XP case reports is different from the general population for non-skin cancers (https://seer.cancer.gov). The most frequent are HEM (34%), CNS (16%), gynecological (13%) and thyroid (9%) tumors while in the general population these tumors only represent 8.9%, 1.3%, 9.8% and 3.3% of all tumors, respectively (Additional file 1: Figure S4A). This distribution is very similar to the one shown for the 4 international XP cohorts (Fig. 1A) indicating there is no distribution bias between the analyzed XP patients.

**Fig. 2:**
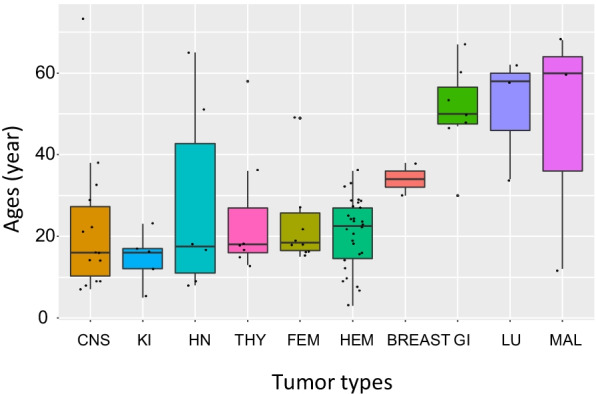
Ages of diagnosis (when known) of internal tumors in all reported XP patients grouped by tumor types. Each black point represents the age at diagnosis for one XP patient with internal tumor. CNS: central nervous system; KI: kidney; HN: head and neck; THY thyroid; FEM: female-reproductive system related tumor; HEM: hematological malignancies; GI: gastro-intestinal; LU: lung; MAL: male-related tumor. The unique Fibrohistiocytoma is not indicated here

The median age at diagnosis of internal tumors in XP patients was significantly lower than in general population, 21 years vs. ~ 65 years, respectively (Additional file 1: Table S4) (Mann–Whitney-Wilcoxon two-sided Test *p*-value < 2.2E−16). The age at diagnosis of internal tumors in XPs varied between tumor types (Fig. 2). The median ages at diagnosis are 15 years for CNS tumors (range: 7–38) and 22.5 years for HEM (range: 3–36). Similar median ages are found for thyroid, head & neck, kidney and gynecological tumors, while patients developing lung (58y), urological (60y) and digestive (50y) tumors are substantially older (Fig. 2 and Additional file 1: Table S4). For example, a 28-years difference of median ages at diagnosis is observed between hematological malignancies and digestive tumors in XP patients (*P* = 1.6E−5 *X*^*2*^ test, Additional file 1: Figure S5).

Internal tumors are frequently lethal in XP patients (Additional file 1: Figure S4B). The median age of death is 25 years. For the patients with HEM the delay between diagnosis and death is an average of 2 years (range: < 1–10 years) and for the CNS tumors, the average time is 1.5 years (range: 1–9 years).

Among the 65 patients, for whom the mutated XP gene was identified, the XP-C group is the most frequent (83%), followed by XP-V (9%) and XP-A (3%) (Additional file 1: Table S2). Tumor types occurring at early ages in XP are almost exclusively associated with the XP-C group: the hematological malignancies (92% are XP-Cs), CNS tumors (90%), gynecological tumors (100%) and thyroid cancers (86%). However, this trend is not observed for digestive and urological cancers associated with later onset in XP (Additional file 1: Table S4).

In this studied collection of internal XP tumors, there were 37 (38 tumors) patients with identified delTG mutation representing 42% of the whole cohort and 69% of the XP-C patients (Additional file 1: Table S2). At the same time, hematological malignancies, gynecological and thyroid tumors in delTG patients represented 81%, 80% and 71% of known complementation groups, confirming the increased risk of these malignancies in the XP-C delTG patients as compared to the remaining XP cohort. The highest risk for XP-C patients with another germline mutation than delTG is for CNS tumors (Additional file 1: Table S4).

## Discussion

To calculate the risk factors of XP patients to develop internal tumors, we made use of 4 very-well characterized XP cohorts from different countries and different laboratories. The Odds ratio for all classes of internal tumors is 34 for the pooled 4 XP cohorts and can go as high as 331 folds for CNS, tumors, 120 folds for HEM and to more than 600 folds for XP patients aged up to 20 years old (Table 2). We calculated the risk factors by comparison with the American general population because the American XP patients represent 32% of the combined cohorts and the American registry of cancer incidence (SEER database) is very comprehensive and well-maintained. Therefore, this use of cancer incidence statistics of the American general population, as a control group for non-US XP cohorts, might cause biases in the estimates of their relative cancer risk as the corresponding populations might be different from the US-population by susceptibility to some cancer types.

Several transgenic mice with germline mutations on XP genes have been constructed. Interestingly enough, internal tumors such as liver, lung cancers or lymphomas are frequently developed in these different XP-mouse models confirming the role of NER deficiency in internal cancers [46] (Supporting Methods and Additional file 1: Table S5) and corroborate human findings.

Among the 4-analyzed XP cohorts, the French one is predominantly associated with one founder mutation in the *XPC* gene which makes it a homogeneous cohort. The reported delTG mutation gives rise to a stop codon and a total absence of the XPC protein, as already described [13]. However, most of the reported *XPC* mutations also give rise to stop codons and did not appear to be associated with a high level of internal cancers. So, a combination of this specific mutation and ethnicity of the patients should be involved in their high risk to develop internal tumors.

Predisposition to hematological malignancies was one of the most pronounced features in young XP patients. The types of hematological malignancies in XP were different from typical sporadic MDS/AML but resembled tumors in patients previously-treated by chemotherapy for a first cancer [42], although the young XP patients were not treated by any anti-tumoral protocol before tumor diagnosis. This similarity suggests that young XPs have rapidly accumulated spontaneous DNA lesions that were not repaired due to NER deficiency. We have recently reported that the mutation load was more than 25-fold higher in XP-C hematological malignancies than in the same tumor types in the general population with a very strong bias toward mutations located on non-transcribed strands [18]. The somatic mutations found in XP-C leukemias closely resemble COSMIC signature 8 [18, 47] that suggests the presence of unrepaired spontaneous purine DNA lesions probably induced by an endogenous oxidative process [18, 29, 48–51].

The high predisposition to hematological malignancies of XP-C patients carrying the North-African germline mutation is also observed in the American cohort (Odds ratio: 74; *p* value = 7.0 E−14; Fisher exact two-sided test) and remains unexplained. Whole exome sequencing of DNA in several patients and their parents did not show any additional pathogenic DNA variants that could be potentially implicated in predisposition to leukemia [42]. We thoroughly searched for additional modifier variants in the genomes and exomes of delTG patients with leukemia (see Additional file 1: Supporting Methods). We identified only one Identical by Descent Segment in all patients. The region of intersection spans 1.02 Mb and includes 13 genes including *XPC*. Besides delTG mutations there were no common or unique pathogenic mutations in these genes (Additional file 1: Figure S6). This analysis reduces the possibility that a common genetic variant in Mediterranean XP-C patients with delTG mutation be responsible for increased risk of leukemia in this cohort.

The patients’ lifestyle with North-African traditions and food may be exacerbating potential internal DNA damage [9, 13]. For example, charcoaled foods used in North Africa are known to produce genotoxic molecules, such as Acrolein that leads to exocyclic mutagenic dG damage [52]. Additionally, the powerful mutagen Aflatoxin B1, known to induce DNA lesions at Gs that are repaired by NER [48], is present in the food in Africa and induces liver cancers. It is plausible that other types of genotoxic contaminants in food from North Africa might be carcinogenic for XP-C patients. Another possibility is that sun-exposure induces some kind of general oxidative stress that will produce DNA lesions not repaired by delTG XP-C patients. Indeed, the XPC protein has also been involved in other DNA repair pathways such as Base Excision Repair [12, 49].

## Conclusion

It is essential that the physicians who treat XP patients be aware of this strong predisposition since XP patients now live longer due to better sun-protection and better knowledge of the disease [53]. The importance of early diagnosis of CNS, thyroid and gynecological cancers and HEM cannot be over-emphasized. The MDS/AML occurring in XP-C patients often appeared following several years of anemia [42] that should be searched for by a regular annual blood analysis starting around the age of 10. Regular gynecological exams and thyroid echography are easy to perform on a regular basis.

## Supplementary Information

**Additional file 1**: **Table S1**. Distribution of internal tumors of all reported XP patients according to organs, complementation groups, ages at diagnosis and death **Table S2**. Distribution of the complementation groups of all reported XP patients with internal tumors **Table S3**. Countries of familial origins of all reported XP patients with internal tumors **Table S4**. Characteristics of internal tumors according to the complementation group of all reported XP patients. **Table S5.** Risk (OR) of internal tumors in mouse XP gene-knockout experiments without exogeneous mutagens **Fig. S1**. Percent of XP patients with internal tumors in the 4 independent XP cohorts **Fig. S2**. Odds Ratio for internal tumor risk in XP patients as compared to the American general population stratified by each individual cohort, tumor types and patient ages. **Fig. S3**. Odds Ratio for internal tumor risk in XP patients as compared to the American general population stratified by complementation groups, tumor types and reproductive system-related tumors. **Fig. S4**. Relative frequencies of XP internal tumor, tumor occurrence and survival in all reported XP patients. **Fig. S5**. Probability of the absence of internal tumors in XP patients stratified between digestive cancers and hematological malignancies. **Fig. S6**. Analysis of the common haplotypes in XP-C delTG patients with leukemia. **Supporting References**.

## Data Availability

The authors confirm that the data supporting the findings of this study are available within the article and its Additional files.
