# Supplementary material for: Increased risk of internal tumors in DNA repair-deficient xeroderma pigmentosum patients: analysis of four international cohorts
Source: Orphanet J Rare Dis. 2022 Mar 4;17:104. doi: 10.1186/s13023-022-02203-1 (PMC8896305; doi:10.1186/s13023-022-02203-1)
Supplement: Supplementary file 1 — Additional file 1: Table S1. Distribution of internal tumors of all reported XP patients according to organs, complementation groups, ages at diagnosis and death Table S2. Distribution of the complementation groups of all reported XP patients with internal tumors Table S3. Countries of familial origins of all reported XP patients with internal tumors Table S4. Characteristics of internal tumors according to the complementation group of all reported XP patients. Table S5. Risk (OR) of internal tumors in mouse XP gene-knockout experiments without exogeneous mutagens Fig. S1. Percent of XP patients with internal tumors in the 4 independent XP cohorts Fig. S2. Odds Ratio for internal tumor risk in XP patients as compared to the American general population stratified by each individual cohort, tumor types and patient ages. Fig. S3. Odds Ratio for internal tumor risk in XP patients as compared to the American general population stratified by complementation groups, tumor types and reproductive system-related tumors. Fig. S4. Relative frequencies of XP internal tumor, tumor occurrence and survival in all reported XP patients. Fig. S5. Probability of the absence of internal tumors in XP patients stratified between digestive cancers and hematological malignancies. Fig. S6. Analysis of the common haplotypes in XP-C delTG patients with leukemia. Supporting References. [file 13023_2022_2203_MOESM1_ESM.docx]

**Additional file 1 for**

**Increased Risk of Internal Tumors in DNA Repair-Deficient Xeroderma Pigmentosum Patients: Analysis of four international cohorts**

**Nikolaev S, Yurchenko A, Sarasin A.**

**Corresponding author:** Alain Sarasin, PhD

Laboratory of Genome Integrity and Cancers, CNRS UMR9019, Gustave Roussy Institute, University Paris-Saclay, Villejuif, France

[alain.sarasin@gustaveroussy.fr](mailto:alain.sarasin@gustaveroussy.fr)

**This PDF file includes:**

**Supporting Methods.**

**Table S1.** Distribution of internal tumors of all reported XP patients according to organs, complementation groups, ages at diagnosis and death

**Table S2.** Distribution of the complementation groups of all reported XP patients with internal tumors

**Table S3.** Countries of familial origins of all reported XP patients with internal tumors

**Table S4.** Characteristics of internal tumors according to the complementation group of all reported XP patients

**Table S5.** Risk (OR) of internal tumors in mouse XP gene-knockout experiments without exogeneous mutagens

**Fig. S1.** Percent of XP patients with internal tumors in the 4 independent XP cohorts

**Fig. S2.** Odds Ratio for internal tumor risk in XP patients as compared to the American general population stratified by each individual cohort, tumor types and patient ages.

**Fig. S3.** Odds Ratio for internal tumor risk in XP patients as compared to the American general population stratified by complementation groups, tumor types and reproductive system-related tumors.

**Fig. S4.** Relative frequencies of XP internal tumor, tumor occurrence and survival in all reported XP patients.

**Fig. S5**. Probability of the absence of internal tumors in XP patients stratified between digestive cancers and hematological malignancies.

**Fig. S6**. Analysis of the common haplotypes in XP-C delTG patients with leukemia.

**Supporting References**

**Supporting Methods**

***Statistical analysis***

Kaplan-Meier estimates were used to compare survival or no tumor appearance between different patient groups (<https://biostatgv.sentiweb.fr/?module=tests/surv>)[1]. Results with P< 0.05 were considered as significant. The references for the distribution and incidence of cancer patients in the French population are described in the legend of the Supplementary Table S6. The statistics of cancer incidence in general population was obtained from the Surveillance, Epidemiology, and End Results (SEER) Program (<https://seer.cancer.gov/>). Skin cancers were excluded from consideration and tumors of the reproductive systems were treated separately according to the gender information in the SEER Program. Odds Ratios and a 95% confidence interval for the Odds Ratios were computed using calcOddsRatio (mymatrix, alpha=0.05) function in R [2]. P-values and 95%CI were indicated in the main text if not available in figures and tables.

***Exome sequencing and analysis***

We sequenced available material from 8 unrelated families of XP-C patients, which included 6 exomes of XP-C patients with leukemia, 6 exomes of their parents (for 3 families), 2 exomes of their XP-C siblings without leukemia (2 families) and 2 exomes of unrelated XP-C patients without leukemia. The exomes were enriched using Agilent V5 capture kit and sequenced on Illumina Hiseq 2500 to 90X mean coverage (Institut Gustave Roussy, France). We aligned the raw reads using BWA mem software [3] and sorted resulted files with the Samtools [4]. The genetic variants were called using GATK Best Practice pipeline for germline exome variants [5,6]. To identify Identical By Descent (IBD) regions among the affected individuals first we phased all the exomes simultaneously with SHAPEIT2 software [7] exploiting familial information (--duohmm) and human recombination map. Then the common IBD regions between individuals were identified using refinedIBD software [8] package and merged with Bedtools [9].

***Mouse models for XP patients***

To get further insights into increased risk of cancer in an XP context, we searched the Mouse Genome Informatics database [10] for mouse homologs of XP genes (*XPA-XPG, POLH*) linked to phenotype “increased tumor incidence” and then reviewed literature choosing only sporadic tumors of internal organs without exogenous toxin application. In total we found eleven experiments summarized in seven studies which used Xpa mice (equivalent of human XP-A), Xpc mice (XP-C) or Ddb2 (XP-E) mice and monitored spontaneous accumulation of tumors with further complex (several organs) or partial (ex. only lungs) pathological examination (see Table S5).

**Table S1.** Distribution of internal tumors of all reported XP patients according to organs, complementation groups, ages at diagnosis and death

| Tumor types  (patient’code) | Complem.  groups | Age at | (years) | Tumor types  (patient’code) | Complem.  groups | Age at | (years) |
| --- | --- | --- | --- | --- | --- | --- | --- |
|  |  | diagnosis | death |  |  | diagnosis | death |
|  |  |  |  |  |  |  |  |
| **Hematology** | **(30/89 =** | **33.7%)** |  | **Digestive (GI)** | **(7/89 =** | **7.9%)** |  |
|  |  |  |  |  |  |  |  |
| Myeloid leukemia | Med Basin* | 32 | 35 | Pancreatic Ad | ? | 47 | NR |
| ALL | XP-A | 3 | 6 | Gastric Ca | ? | 67 | NR |
| RAEB (XPGaAiVI) | delTG† | 24 | 25 | Gastric Ad | ? | 30 | 31 |
| RAEB (XPGaMVI) | delTG | 27 | 27 | Bile duct Ca | XP-F | 60 | 65 |
| Aplastic anemia | XP-C | 10 | NR | Stomach Ca | XP-V | 53 | 53 |
| Lymphatic leukemia | Med Basin | 16 | 18 | Gastric Ad  (XPGAVI) | XP-V | 48 | 54 |
| T-cell lymphoma (XP04SP) | delTG | 3 | 13 | Gastric Ad  (2P0) | XP-V | 50 | 54 |
| T-ALL & AML-6 (XP924VI) | delTG | 12 | 15 |  |  |  |  |
| Megakaryoblastic leukemia | XP-D | 33 | 34 | **Thyroid** | **(8/89 =** | **9%)** |  |
| Leukemia | delTG | 9 | 10 |  |  |  |  |
| RAEB/AML | Med Basin | 28 | 28 | Neuroendocrine Tu (XP148VI) | delTG | 18 | 19 |
| ALL | Med Basin | 22 | 25 | Follicular Ca | Med Basin | 17 | NR |
| AML-4 (XP10VI) | delTG | 27 | 28 | Papillary Ca (XP802VI) | delTG | 18 | NR |
| AML-6 (XP82VI) | delTG | 16 | 18 | Thyroid Ca | delTG | 13 | 15 |
| AML-6 (XP235VI) | delTG | 24 | 29 | Thyroid Ca | delTG | 15 | 29 |
| B-ALL & MDS (XP309VI) | delTG | 7 | 10 | Thyroid Ca (XPAAVI)  Thyroid Ca  Thyroid Ca | delTG  XP-C  XP-E | 17  58  36 | Alive  Alive  Alive |
| AML (XP185VI) | delTG | 24 | 25 |  |  |  |  |
| RAEB-t (XP167VI) | delTG | 25 | 26 | **Head & Neck** | **(6/89 =** | **6.7%)** |  |
| AML (XPAHVI) | delTG | 23 | 25 |  |  |  |  |
| T-ALL (XP673VI) | delTG | 21 | 22 | Throat Ca | ? | 65 | NR |
| AML (XP538VI) | delTG | 29 | 29 | Gingival SC | ? | 9 | NR |
| AML-6 (XP2006VI) | delTG | 29 | Alive | Gingival Tu | ? | 17 | NR |
| B-cell lymphoma (XP393BE) | delTG | 29 | 29 | Palate SC | ? | 18 | NR |
| MPAL (XP540BE) | delTG | 19 | Alive | Pharynx SC | XP-V | 51 | 56 |
| MDS/AML (XP30BE) | HZ delTG‡  HZ *XPC* | 36 | 38 | Gingival SC | XP-C | 8 | 10 |
| MDS (XP243BE) | XP-C | 18 | 20 |  |  |  |  |
| AML-M2$ (XP2004VI) | delTG | 22 | 23 | **Kidney** | **(5/89 =** | **5.6%)** |  |
| T-lymphoma (XP208VI) | delTG | 8 | 17 |  |  |  |  |
| AML-3 (XPMaAbVI) | delTG | 14 | Alive | Renal Lei | ? | 12 | 13 |
| NK lymphoma (XP420VI) | delTG | 24 | 25 | Wilm’s Tu | Med Basin | 17 | 18 |
|  |  |  |  | Wilm’s Tu | Med Basin | 16 | 17 |

**Table S1.** Distribution of internal tumors of XP patients according to organs, complementation groups, ages at diagnosis and death (continued)

| **CNS** | **(14/89 =** | **15.7%)** |  | Kidney Ad (XP165VI) | delTG | 23 | 25 |
| --- | --- | --- | --- | --- | --- | --- | --- |
|  |  |  |  | Nephroblastoma | Med Basin | 5 | NR |
| Astrocytoma | ? | 9 | NR |  |  |  |  |
| Medulloblastoma | ? | 14 | NR | **Urology (MAL)** | **(3/89 =** | **3.4%)** |  |
| Brain sarcoma | ? | 16 | NR |  |  |  |  |
| Brain sarcoma | ? | 33 | 35 | Testicular Tu | ? | 12 | NR |
| Glioblastoma | XP-A | 8 | 9 | Bladder Ca | XP-V | 68 | 68 |
| Astrocytoma (XP233VI) | delTG | 7 | 8 | Prostate Ca (XP819VI) | XP-V | 60 | alive |
| Glioblastoma (XP15BE) | XP-C | NR | 16 |  |  |  |  |
| Astrocytoma (XP23BE) | XP-C | 22 | 31 | **Lung** | **(3/89 =** | **3.4%)** |  |
| Glioblastoma (XP24BE) | XP-C | 29 | 35 |  |  |  |  |
| Schwannoma (XP14BE) | XP-C | NR | 73 | Bronchogenic Ca (XP3BE) | XP-C | 34 | 37 |
| Astrocytoma (XP664VI) | delTG | 9 | 10 | Bronchogenic Ca  Lung Ca | XP-C  XP-C | 62  58 | NR  NR |
| Neuroepithelial Tu (XP28BR) | XP-C | 21 | NR |  |  |  |  |
| Glioblastoma (XP21BR) | XP-C | 38 | 39 | **Fibrohistio-cytoma** | **(1/89 =** | **1.1%)** |  |
| Astrocytoma (XPAdSaVI) | delTG | 14 | 19 |  |  |  |  |
|  |  |  |  | Fibrohistio- cytoma | XP-C | 20 | NR |
| **Gynecology**  **Breast** | **(12/89 =** | **13.5%)** |  |  |  |  |  |
| Breast Ca | ? | 38 | NR |  |  |  |  |
| Breast Ca  (XPMYVI)  **FEM** | XP-C | 30 | 30 |  |  |  |  |
| Uterine Ca | ? | 49 | 51 |  |  |  |  |
| Uterine Ad (XP1BE) | XP-C | NR | 49 |  |  |  |  |
| Cervical Sa (XP269VI) | delTG | 18 | 23 |  |  |  |  |
| Uterine Lei | delTG | 19 | alive |  |  |  |  |
| Uterine Lei | delTG | 28 | 29 |  |  |  |  |
| Ovary Ca (XP19P0) | delTG | 27 | alive |  |  |  |  |
| Uterine Rhab$ (XP2004VI) | delTG | 16 | 23 |  |  |  |  |
|  |  |  |  |  |  |  |  |
| Uterine Rhab  (XP2003VI) | delTG | 16 | Alive |  |  |  |  |
| Ovarian Sa (XPElHaVI) | delTG | 18 | 22 |  |  |  |  |
| Uterine Ad (XPElKaVI) | delTG | 15 | alive |  |  |  |  |

Abbreviations: NR, not reported in the publications; Ca, carcinoma; Tu, tumor; Ad, adenocarcinoma; Sa, sarcoma; Lei, leiomyosarcoma; Rhab, rhabdomyosarcoma; SC, squamous cell; FEM, female-reproductive system related tumor; MAL, male-reproductive system related tumor.

*These XP patients are living in a country around the south coast of the Mediterranean Sea and are probably XP-C with the North-African founder mutation (delTG, see below). We called them “Med Basin”

†delTG means XP-C patients with the North-African founder mutation: c.1643_1644delTG; p.Val548AlafsX572 [11].

‡This XP-C patient was a compound heterozygous with one allele delTG [12].

$This patient had developed two different unrelated internal tumors [13].

**Table S2.** Distribution of the complementation groups of all reported XP patients with internal tumors

| XP groups | Patient No.* | Male† | Female† | *XPC* delTG‡ |
| --- | --- | --- | --- | --- |
| XP-A | 2 (2%) | 1 | 1 |  |
| XP-C | 54 (61%) | 23 | 28 | 37 (42 %) |
| XP-D | 1 (1%) | 1 | 0 |  |
| XP-E | 1 (1%) | 0 | 1 |  |
| XP-F | 1 (1%) | 0 | 1 |  |
| XP-V | 6 (7%) | 5 | 1 |  |
| Unknown | 23 (26%) | 12 | 11 |  |
|  |  |  |  |  |
| Total | 88 | 42 | 43 |  |

*One patient had two independent tumors. We have 88 patients for 89 tumors. The % of patients is given as compared to the entire XP population, including those with unknown complementation group.

†When known.

‡This mutation corresponds to the homozygous founder mutation in North Africa: *XPC* c.1643_1644delTG; p.Val548AlafsX572 described in Soufir *et al*.[11]^.^

**Table S3.** Countries of familial origins of all reported XP patients with internal tumors

*One patient had two independent tumors: the number of patients is 88 for 89 total tumors

†*XPC* delTG refers to the founder mutation found in the vast majority of XP-C patients from North Africa: c.1643_1744delTG; p.Val548AlafsX572 [11].

‡One patient was reported to be originated from North Africa without precision on his country [12].

$These patients have been reported in the NIH (National Institute of Health, Bethesda, USA) studies from the Kraemer’s group, but the countries of origin are not always indicated. So, we classified these patients as Americans. However, two of them described in the United States are indeed originated from North Africa and Morocco and one XP-C patient is heterozygous for the delTG mutation [12].

**Table S4.** Characteristics of internal tumors according to the complementation group of all reported XP patient

**
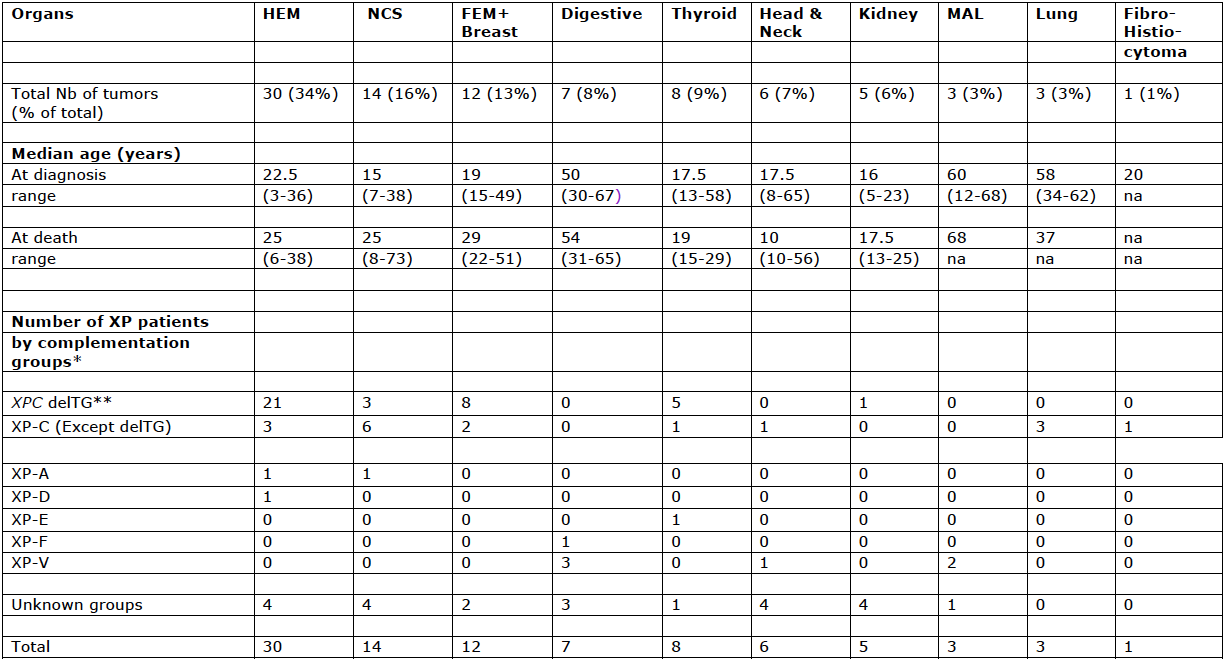
**

Abbreviations: na, not applicable; HEM: hematology; NCS: nervous central system; FEM: female reproductive system related tumor; MAL: male reproductive system related tumor.

*A unique patient (XP2004VI) had two independent internal tumors (gynecology and hematology [13]) and is counted twice here.

**delTG corresponds to the North African founder mutation: *XPC* c.1643_1644delTG; p.Val548AlafsX572 [11].

**Table S5.** Risk (OR) of internal tumors in mouse XP gene-knockout experiments without exogeneous mutagens

*The pituitary tumor was a benign neoplasm [14].

**Fig. S1.** Percent of XP patients with internal tumors in the 4 independent XP cohorts

The 4 cohorts are the French (F-XP), the American (A-XP), the Brazilian (B-XP) and the English (UK-XP) ones. The overall average of patients with internal tumor is 11.3%. The red numbers indicate the raw number of XP patients with internal tumor. One French patient had two different internal tumors.
In the combined 4 cohorts, the relative risk of internal tumors is 34 times higher than in the general population (“gen_pop”) (p-value= 1.0E-47).

14

2

31

2

0

5

10

15

F-XP

XP cohorts

% of XP patients with internal tumors

F-XP

A-XP

B-XP

UK-XP

gen_pop

Average 11.3%

A-XP

B-XP

UK-XP

Gen_pop

**Fig. S2.** Odds Ratio for internal tumor risk in XP patients as compared to the American general population stratified by each individual cohort, tumor types and patient ages.

The 4 independent XP cohorts are A (or A-XP) for USA, B (or B-XP) for Brazil, UK (or UK-XP) for United Kingdom and F (or F-XP) for France. A) represents the risk for the whole cohort except for the tumors of the reproductive system that are treated separately. The numbers in red indicate the Odds ratio: 56 (95% CI= 37 to 84) for F-XP; 33 (19 to 59) for A-XP; 7 (1.8 to 30) for UK-XP and 10 (1.4 to 73) for B-XP. B) represents the risk according to the types of internal tumors except for the tumors of the reproductive system. C) represents the risk according to the age classes of patients except for the tumors of the reproductive system.

CNS: central nervous system; HEM: hematological malignancies; THY: thyroid; LU: lung; GI: gastro-intestinal; KI: kidney tumors.

**Fig. S3.** Odds Ratio for internal tumor risk in XP patients as compared to the American general population stratified by complementation groups, tumor types and reproductive system-related tumors.

Same legend as in Fig. S2.

**Fig. S4.** Relative frequencies of XP internal tumor, tumor occurrence and survival in all reported XP patients.

A

B

A) Proportion of tumor types, excluding skin cancers, in the XP population (red) as compared to the general American population (blue). The raw counts of XP tumors reported in this paper are indicated in red (the unique fibrohistiocytoma tumor is not inserted in this figure). Tumor type distribution for the American general population (blue) is computed using (SEER) Program ([https://seer.cancer.gov/](https://mail.gustaveroussy.fr/owa/redir.aspx?C=gfpgsKGBIbd4xAI6rqvsILZkDXRJIL4JWTRGLOQ4WlA60Y_HWVrYCA..&URL=https%3a%2f%2fseer.cancer.gov%2f)) data. CNS: central nervous system; FEM: female-reproductive system related tumor; GI: gastro-intestinal; HEM: hematological malignancies; HN: head and neck; KI: kidney; LU: lung; MAL: male-reproductive system related tumor; THY thyroid.

B) Internal tumor occurrence and survival in XP patients. Blue curve: Probability of the absence of internal tumors in 83 patients with a median age at diagnosis of 21 years. Red curve: Kaplan-Meier distribution of XP survival for 86 patients with a median age at death of 25 years. The numbers of represented tumors are different from the 89 reported tumors in Table 3 and Table S1 because we don’t have full information for all of them.

**Fig. S5.** Probability of the absence of internal tumors in XP patients stratified between digestive cancers and hematological malignancies


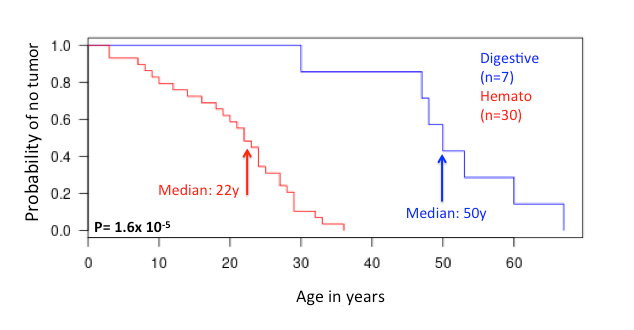


Seven XP patients with digestive cancers (blue curve) and 30 XP patients with hematological malignancies (red curve). Median ages at diagnosis were 22 years for hematological and 50 years for digestive tumors (P=1.6E-5; *X*^2^ test).

**Fig. S6**. Analysis of the common haplotypes in XP-C delTG patients with leukemia.

A) Pairwise comparison of identical by descent haplotypes between patients. X–axis represents the chromosome numbers and the Y-axis the percentage of patients sharing the same haplotype. B) Zoom of panel A to the region of the only haplotype which is identical among all patients and includes the *XPC* gene (1.02 Mbp long on chr. 3). C) UCSC browser snapshot of the Identical by Descent Region between all the studied patients.

**Supporting References**

**1.** Kassambara, A., Kosinski, M., Biecek, P., Fabian, S. Survminer: Drawing Survival Curves using'ggplot2'. R package version 0.3. **1**, (2017).

**2**.  Avril Coghlan. A Little Book of R for Biomedical Statistics Release 0.2, Wellcome Trust Sanger Institute, Cambridge, UK. March 23, (2015).

**3.** Li, H, & Durbin, R. Fast and accurate long-read alignment with Burrows–Wheeler transform. *Bioinformatics* **25**, 1754-1760 (2009).

**4.** Li, H. et al. The sequence alignment/map format and SAMtools. *Bioinformatics* **25**, 2078-2079 (2009).

**5.** Van der Auwera, G. A. From FastQ data to high‐confidence variant calls: The Genome Analysis Toolkit best practices pipeline. *Curr. Protoc. Bioinformatics* **11**, 11.10.1-11.10.33 (2013).

**6.** McKenna, A. et al. The Genome Analysis Toolkit: a MapReduce framework for analyzing next-generation DNA sequencing data. *Genome Res.* **20**, 1297-1303 (2010).

**7.** Delaneau O. et al. Improved whole-chromosome phasing for disease and population genetic studies. *Nat. Methods* **10**, 5-6 (2013).

**8**. Browning, B. L. & Browning, S. R. Improving the accuracy and efficiency of identity-by-descent detection in population data. *Genetics* **194**, 459-471 (2013).

**9.** Quinlan, A. R. BEDTools: the Swiss‐army tool for genome feature analysis. *Curr. Protoc. Bioinformatics*  **47,** 11.12.1-11.12.34 (2014).

**10.** Eppig, J. T. Mouse genome informatics (MGI) resource: Genetic, Genomic, and Biological Knowledgebase for the laboratory mouse. *ILAR J.* **58**, 17-41 (2017).

**11**. Soufir, N. et al. A prevalent mutation with founder effect in xeroderma pigmentosum group C from North Africa. *J. Invest. Dermatol*. **130,** 1537-1542 (2010).

**12**. Oetjen, K.A. et al. Predisposition to hematologic malignancies in patients with xeroderma pigmentosum. *Haematologica.* **104**, 223370 (2019).

**13**. Yurchenko, A. A. et al. XPC deficiency increases risk of hematologic malignancies through mutator phenotype and characteristic mutational signature. *Nat. Comm*. 11, 5834 (2020).

**14.** Melis, J. P. et al. Mouse models for xeroderma pigmentosum group A and group C show divergent cancer phenotypes. *Cancer Res*. 68, 1347-1353 (2008).

**15**. Takahashi, Y. et al. Enhanced spontaneous and aflatoxin-induced liver tumorigenesis in xeroderma pigmentosum group A gene-deficient mice. *Carcinogenesis,* 23, 627-633 (2002).

**16.** Nakane, H. et al. Impaired spermatogenesis and elevated spontaneous tumorigenesis in xeroderma pigmentosum group A gene (Xpa)-deficient mice. *DNA repair (Amst),* **7,** 1938-1950 (2008).

**17**. Hollander, M. C. et al. Deletion of *XPC* leads to lung tumors in mice and is associated with early events in human lung carcinogenesis. *Proc. Natl. Acad. Sci. USA,* **102**, 13200-13205 (2005).

**18.** Zhang, X. et al. Genetic evidence for XPC-KRAS interactions during lung cancer development. *J. Genet. Genomics,* **42,** 589-596 (2015).

**19**. Yoon, T., Chakrabortty, A., Franks, R., Valli, T., Kiyokawa, H. & Raychaudhuri, P. Tumor-prone phenotype of the DDB2-deficient mice. *Oncogene,* **24**, 469-478 (2005).

**20.** Itoh, T. et al. Ddb2 is a haploinsufficient tumor suppressor and controls spontaneous germ cell apoptosis. *Hum. Molec. Genetics,* 16, 1578-1586 (2007).
